# Effect of bovine pericardial extracellular matrix scaffold niche on seeded human mesenchymal stem cell function

**DOI:** 10.1038/srep37089

**Published:** 2016-11-15

**Authors:** Zhi Zhao Liu, Maelene L. Wong, Leigh G. Griffiths

**Affiliations:** 1 Department of Veterinary Medicine: Medicine and Epidemiology, University of California, Davis, One Shields Ave., Davis, CA 95616, USA

## Abstract

Numerous studies have focused on generation of unfixed bovine pericardium (BP) extracellular matrix (ECM) for clinical application. However, the extent to which maintenance of native ECM niche is capable of directing behavior of repopulating cells remains relatively unexplored. By exploiting the sidedness of BP scaffolds (i.e., serous or fibrous surface), this study aims to determine the effect of ECM niche preservation on cellular repopulation using different scaffold generation methods. BP underwent either sodium dodecyl sulfate (SDS) decellularization or stepwise, solubilization-based antigen removal using amidosulfobetaine-14 (ASB-14). SDS scaffolds were toxic to repopulating human mesenchymal stem cells (hMSC). Scanning electron microscopy revealed distinct surface ultrastructure of ASB-14 scaffolds based on native BP sidedness. Basement membrane structures on the serous side stimulated hMSC cell monolayer formation, whereas fibrous side facilitated cell penetration into scaffold. Additionally, serous side seeding significantly increased hMSC adhesion and proliferation rate compared to the fibrous side. Furthermore, scaffold ECM niche stimulated sidedness dependent differential hMSC human leukocyte antigen expression, angiogenic and inflammatory cytokine secretion. This work demonstrates that ECM scaffold preparation method and preservation of BP side-based niches critically affects *in vitro* cell growth patterns and behavior, which has implications for use of such ECM biomaterials in clinical practice.

Bovine pericardium (BP)-derived biomaterials, first introduced into clinical practice in bioprosthetic heart valves^1^, are now widely used^2^,^3^,^4^. Overcoming the aggressive recipient graft-specific rejection response is critical to utilizing such xenogeneic biomaterials in clinical practice^5^. Although glutaraldehyde fixation prevents hyperacute and acute immune responses towards BP by masking xenoantigens from immediate recognition, persistence of xenoantigens in the biomaterial elicits chronic immune-mediated degeneration and subsequent calcification^6^,^7^,^8^. Furthermore, glutaraldehyde-fixed BP is incompatible with constructive recipient cellular repopulation^9^,^10^, limiting biomaterial regenerative potential. Unfixed extracellular matrix (ECM) scaffolds in which antigenicity has been reduced or eliminated have potential to overcome the limitations of current glutaraldehyde-fixed BP, further expanding the use of this important biomaterial in clinical practice.

A potentially ideal BP ECM scaffold should exhibit reduced antigenicity, while maintaining structure-function properties and recellularization potential^11^,^12^,^13^. A range of approaches have been explored for reducing antigenicity of BP including decellularization (e.g., SDS, Triton X-100, trypsin)^14^,^15^, enzymatic removal of α-Gal (i.e., α-galactosidase)^16^, and stepwise, solubilization-based antigen removal^17^,^18^. Although reports of complete acellularity following SDS-decellularization have made this approach a literature gold standard, significant alterations in ECM structure-function properties resulting from denaturation by this harsh ionic detergent have been widely reported^15^,^16^,^18^. Conversely, stepwise, solubilization-based antigen removal has been demonstrated to significantly reduce BP antigenicity while maintaining native tissue structure-function properties^18^. However, the extent to which preservation of native ECM niche is capable of directing behavior of repopulating cells remains relatively unexplored.

Mesenchymal stem cells are the focus of intense investigation for regenerative medicine applications due to their immunomodulatory and pro-regenerative properties^19^,^20^,^21^. Human mesenchymal stem cells (hMSC) are a self-renewing cell population that retains the ability to differentiate into multiple downstream lineages^22^,^23^,^24^,^25^. hMSC exhibit immunomodulatory effects via both direct cell-to-cell contact and indirect cytokine secretion mechanisms^26^,^27^, which in combination with their low surface expression of human leukocyte antigen (HLA)^28^, may confer additive or synergistic benefits in limiting recipient scaffold-specific immune responses. Furthermore, through release of paracrine factors, hMSC exhibit a range of potential pro-regenerative effects, including ability to modulate local angiogenesis, reduce fibrosis, and promote recruitment of endogenous effectors cells^29^,^30^. However, the extent to which ECM scaffold niches modulate hMSC functional parameters remains largely unexplored.

The differential anatomical organization of BP, which includes a specialized ECM basement membrane on the serous side and loose connective tissue on the fibrous side, provides an opportunity to examine cellular behavior in different ECM niches. We hypothesize that both ECM damage and residual toxicity of chemicals used in different scaffold generation methods have the potential to negatively affect cellular repopulation of BP ECM scaffolds. We further hypothesize that in scaffolds with preserved ECM niches, hMSC behavior will differ based on the ECM microenvironment onto which the cells are seeded. In this work, we assess the effect of BP ECM scaffold generation method and microenvironment on resultant hMSC adhesion, migration, proliferation, ECM matrix production, cytokine release (angiogenic and anti-inflammatory factors), and HLA expression patterns.

## Methods

### BP scaffold preparation

Bovine pericardia from adult cattle (Spear Products, Coopersburg, PA) were stripped of pericardial fat and loose connective tissue, trimmed to 0.2 g pieces (approximately 1 cm × 2 cm), and subjected to antigen removal or decellularization as described previously. Briefly, for amidosulfobetaine-14 (ASB-14, Sigma, St. Louis, MO) scaffolds, intact pieces of BP were subjected to hydrophile solubilization buffer (10 mM Tris-HCl pH 8, 0.5 mM Pefabloc, 100 mM DTT, 100 mM KCl, 2 mM MgCl_2_•6H_2_O, 1% (v/v) antibiotic antimycotic solution, Sigma) for 48 h, followed by lipophile solubilization (1% (w/v) ASB-14 in hydrophile solubilization buffer) for 48 h at room temperature^18^. For SDS-decellularized scaffolds, BP pieces were subjected to 0.1% (w/v) SDS (Bio-Rad, Hercules, CA) containing 1% (v/v) antibiotic antimycotic solution for 48 h, followed by 1% (w/v) SDS containing 1% (v/v) antibiotic antimycotic solution for 48 h at room temperature^18^. Both groups were then treated by nucleic acid digestion (10 mM Tris-HCl pH 7.6, 150 mM NaCl, 5 mM MgCl_2_•6H_2_O, 2.5 Kunitz units/mL DNAse I, 7.5 Kunitz units/mL RNAse A, 1% (v/v) antibiotic antimycotic solution) for 24 h, followed by washout in 0.5 mM Pefabloc, 10% (v/v) Tris-Buffered saline, 1% (v/v) antibiotic antimycotic solution for 96 h. All steps of the protocol were performed in 2 mL working volume at 4 °C and 125 rpm unless otherwise stated. All solutions were changed twice per day. BP scaffolds were stored in Dulbecco’s modified Eagle’s medium (Sigma) containing 15% (v/v) dimethyl sulfoxide (Sigma) at −80 °C.

### hMSC isolation and eGFP transduction

All stem cell experiments were performed in accordance with guidelines of the Institutional Stem Cell Research Oversight Committee and Biological Use Authorization regulations of University of California, Davis. hMSC were isolated from fresh, unprocessed bone marrow from three healthy donors (Lonza, Allendale, NJ) and transduced with enhanced green fluorescent protein (eGFP) as previously described^31^. The pCCLc-MNDU3-LUC-PGK-EGFP-WPRE lentiviral vector was generated by the UC Davis Vector Core. To transduce hMSC, cells were plated in a 12-well plate at 5 × 10^4^ cells/well. The following day, lentivirus was added to culture medium at multiplicities of infection of 11 in the presence of 20 μg/ml protamine sulfate (Sigma), used to increase nonspecific viral uptake by cells. hMSC were cultured in culture medium consisted of Dulbecco’s modified Eagle’s medium high glucose (DMEM), 1% (v/v) penicillin-streptomycin (P/S), 1% (v/v) L-glutamine 200 mM (Hyclone Laboratories, South Logan, UT, USA) and 20% (v/v) fetal bovine serum (FBS, Atlanta Biologicals, Lawrenceville, GA, USA). Cells were examined for fluorescence 72 h after initial virus addition and then cryopreserved for use in later experiments. Three hMSC lines were used for qualitative experiments, and a single hMSC line was used for quantitative studies. All experiments utilized hMSC at passage 3–5.

### Detergent cytotoxicity

The MultiTox-Fluor Multiplex Cytotoxicity Assay (Promega, Madison, WI) was used to test the cytotoxicity of ASB-14 and SDS. hMSC were cultured in 96-well black plates (Corning, Corning, NY) at 6,000 cells/well h at 37 °C, 5% CO_2_ for 24 h. A concentration gradient of ASB-14 (0.01%, 0.007%, 0.005%, and 0.0027% w/v) and SDS (0.015%, 0.012%, 0.01%, 0.005% and 0.0015% w/v) were dissolved in culture medium. 100 μL medium spiked with ASB-14 or SDS (n = 6 per group) was added to cell monolayers for 1 h, followed by addition of 100 μL of MultiTox-Fluor Reagent for 1.5 h. Fluorescence intensity was measured using Cytation3 (BioTek, Winooski, VT) plate reader (live-cell fluorescence at 400Ex/505Em; dead-cell fluorescence at 485Ex/520Em). Untreated cells and culture medium served as a vehicle control and background fluorescence, respectively. All samples were run in triplicate.

### Residual BP scaffold detergent cytotoxicity

For washout toxicity experiments, ECM scaffolds were generated with different washout durations (1, 2, 4 and 7 d) applied. Following washout, BP scaffolds (n = 6 per group/time point) were immersed in 2 mL culture medium at 4 °C and 125 rpm overnight, with this final washout media collected and stored at −20 °C for cytotoxicity evaluation. hMSC were cultured in 96-well plates (Corning, Corning, NY) at 30,000 cells/well overnight. The next day, medium in each well was replaced with 250 μL of pre-warmed washout medium. After 2 h incubation on cell monolayers at 37 °C, 5% CO_2_, washout medium was removed and replaced with 100 μL fresh culture medium and 10 μL alamarBlue (Invitrogen, Carlsbad, CA) for an additional 1.5 h. 100 μL of medium from each well was transferred to 96-well plate for measurement of fluorescence intensity using a Cytation3 imaging reader (560Ex/590Em). Untreated cells served as a vehicle control and culture medium served as background fluorescence. All samples were run in triplicate.

### Cell seeding and imaging

All recellullarization studies utilized 6 mm diameter biopsy punches (Acuderm Inc., Fort Lauderdale, FL) to generate discs for each scaffold type, which were placed into 96-well plates. 30,000 eGFP-hMSC were seeded onto either the fibrous or serous side of BP scaffolds in a final culture medium volume of 250 μL. Seeded scaffolds were incubated at 37 °C, 5% CO_2_; culture medium was changed every day. At day 4 post-seeding, eGFP-hMSC seeded on BP scaffolds were imaged at 488Ex/505-545Em (Leica DMI6000 B, Leica Company).

### Immunohistochemistry and immunofluorescence staining

BP scaffolds with/without hMSC seeding were histologically processed using hematoxylin and eosin (H&E), immunohistochemistry, and immunofluorescence double staining. Briefly, the scaffolds were fixed in 10% buffered formalin, embedded in paraffin, and processed for staining. Laminin immunohistochemistry was performed using anti-laminin primary antibody (1:50, Abcam, Cambridge, MA) and horseradish peroxidase (HRP)-conjugated anti-rabbit secondary antibody (DAKO, Carpinteria, CA). Immunofluorescence double staining was performed using mouse anti-eGFP (1:200, Abcam, Cambridge, MA) and rabbit anti-laminin primary antibodies. Fluorescent anti-mouse secondary antibody and anti-rabbit secondary antibody tagged with Alexa Fluor 405 (402Ex/421Em) and Alexa Fluor 647 (652Ex/668Em) (1:500, Abcam), respectively, were used for visualization. For both stains, slides were imaged using a Nikon Eclipse E600 microscopy and digital images collected.

### Scanning electron microscopy (SEM)

ASB-14 scaffolds (n = 4 per group) with or without hMSC seeding on either the serous or fibrous surface were fixed in neutral 10% formalin overnight at room temperature and dehydrated through a graded series of ethanol. After critical-point drying, scaffolds were mounted on aluminum stubs with the cell-loaded surface oriented upwards, sputter coated with gold-palladium (AuPd), and viewed using SEM (Philips XL30 TMP; FEI Company).

### Maximal seeding density and binding kinetics

To determine maximal seeding density, hMSC were seeded onto either the serous or fibrous surface of ASB-14 scaffolds at increasing levels from 10,000 to 90,000 cells per 6 mm scaffold disc (n = 6 per group) in a non-treated 96-well plate (Corning, Corning, NY). AlamarBlue assay was performed after 24 h as described above. The culture medium from scaffolds alone was used for background fluorescence.

To evaluate cell binding kinetics, 30,000 cells/scaffold were seeded onto either the serous or fibrous surface of ASB-14 scaffolds and allowed to adhere to ECM for a range of durations (30 min, 1 h, 2 h, and 4 h, n = 4 per group/time point). Following each time point, scaffolds were placed on orbital shaker 125 rpm at room temperature for 1 min, and then gently washed twice in 250 μL culture medium to remove non-adherent cells. Recellularized scaffolds were incubated for an additional 24 h and alamarBlue assay performed as described above, using 2 h alamarBlue loading time.

### Proliferation

hMSC (at 10,000, 30,000, 60,000, 90,000 cells/scaffold) were seeded onto either the serous or fibrous surface of ASB-14 scaffolds (n = 7 per group/density). Seeded scaffolds were then cultured for 7 d, with alamarBlue assay performed at 1, 3, 5, and 7 d.

### Immunocytology

30,000 hMSC without eGFP labelling were cultured on either the serous or fibrous surface of ASB-14 scaffolds, or tissue culture plastic for 3 d. Cells were harvested by immersion in 0.25% trypsin (Sigma) at 37 °C and 150 rpm for 15 min. Cells were fixed and permeabilized, and then smeared on gelatin-coated slides (FD NeuroTechnologies, Ellicott City, MD). Cells were stained with anti-HLA Class I ABC (1:100, Abcam), HLA Class II DRB1 (1:100, Abcam) and HLA G (1:100, Abcam), separately, and subsequently stained with 1:200 dilution of Alexa Fluor 488 (498Ex/520Em) conjugated anti-mouse or anti-rabbit secondary antibody (Abcam). After mounting with DAPI (Abcam), slides were observed and digital images were collected. The mean fluorescent intensity of at least 20 cells from each group and stain were analyzed by NIS-Element software.

### Tri-lineage differentiation

hMSC were detached from ASB-14 scaffolds after 7 d of culture as described above. Retrieved cells were placed on 6-well plates (Corning, Corning, NY) for expansion to desirable density for differentiation. Adipogenic, osteogenic, and chondrogenic induction (n = 5 per group) using tri-lineage differentiation media (Lonza) were performed according to the manufacturer’s specifications. Cell differentiation was confirmed with Alizarin Red S (Sigma), Oil Red O (Cayman, Ann Arbor, MA), and Alcian Blue staining for osteocytes, adipocytes, and chrondrocytes, respectively.

### Cell supernatant assays

#### Conditioned media collection

Seeded scaffolds (30,000 cells/scaffold) or cells only were cultured in culture medium, and the resultant conditioned medium were collected every 24 h for 3 d and pooled. Time-matched, unseeded scaffolds incubated in culture medium served as a vehicle control. Supernatant were preserved at −80 °C for subsequent experiments.

#### Microarray profiling and ELISA quantification of inflammatory cytokine secretion

150 μL of conditioned medium from each scaffold was pooled (n = 6 per group) and assayed using a Human Inflammatory Array C3 kit (RayBiotech, Norcross, GA) for expression profiles of inflammatory-related proteins according to the manufacturer’s instructions. Array data were captured and analyzed by FluorChem Xplor (Alpha Innotech, San Leandro, CA). Conditioned media (n = 6 per group) was quantified for hMSC monocyte chemoattractant protein-1 (MCP-1) secretion using a Human MCP-1 ELISA kit (RayBiotech) according to the manufacturer’s instructions. Absorbance at 450 nm was measured using a Cytation3 plate reader.

#### Quantitative microarray of angiogenic cytokine secretion

Conditioned media (n = 6 per group) was assayed using a Human Angiogenesis Array Q1 kit (Raybiotech) to determine concentration of angiogenesis-related cytokines. The experiment was performed according to the manufacturer’s instructions. Array data were captured by GenePix 4000B and analyzed by GenePix Pro 6 (Molecular Device).

#### Statistical analysis

All data are expressed as mean ± standard deviation. One-way ANOVA and Tukey’s multiple comparisons post hoc test were performed on cytotoxicity of residual detergents in scaffolds, HLA fluorescent intensity and ELISA. Two-way ANOVA and Tukey’s multiple comparisons post hoc test were performed on hMSC seeding efficiency and kinetics. Repeated measurement two-way ANOVA and Tukey’s multiple comparisons post hoc test were performed on hMSC proliferation. For all analyses *p* < 0.05 was considered to be significant.

## Results

### Relative toxicity of SDS and ASB-14

**Figure 1:**
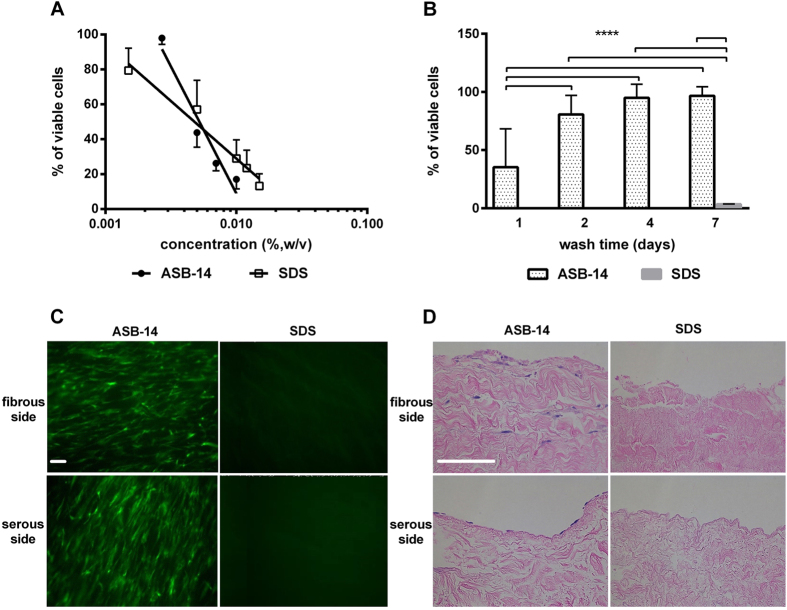
Effect of scaffold generation method on hMSC reseeding. (**A**) Cytotoxicity of SDS versus ASB-14 in solution, demonstrating no difference in LD_50_ between the two compounds (n = 6 per group). (**B**) Cytotoxicity of BP scaffolds with increasing days of washout. ASB-14 scaffolds are non-toxic following 4 d of washout, whereas SDS scaffolds remain toxic to 96.5% of hMSC even after 7d of washout. (**C**) hMSC are observed on both fibrous and serous sides of ASB-14 scaffolds, but not SDS scaffolds (100×). (**D**) Hematoxylin and eosin (H&E) staining of seeded BP scaffolds demonstrates that cells seeded on the fibrous side penetrate into the scaffold, while those seeded on the serous side form a monolayer (200×). Scale bar represents 100 μm. Data are presented as the mean ± standard deviation (****p < 0.001).

No difference was found between the lethal dose to 50% of cells (LD_50_) for ASB-14 (0.0052% w/v) and SDS (0.0048% w/v) (Fig. 1A). Toxicity of ASB-14 scaffolds decreased with increasing wash duration (*p* < 0.001), plateauing at 5.0% toxicity following 4 d washout (Fig. 1B). Toxicity of SDS scaffolds remained at 96.5% even after 7 d washout.

### Effect of scaffold generation method on hMSC seeding

No visible nuclei were detected in either ASB-14 or SDS scaffolds (data not shown). hMSC were present on both fibrous and serous sides of ASB-14 scaffolds after 4 d culture. However, no cells were observed on SDS scaffolds (Fig. 1C). H&E staining shows hMSC formed a cell monolayer on serous side with no infiltration below the ECM surface, while fibrous side seeding resulted in hMSC migration into ASB-14 scaffolds (Fig. 1D). Maximal hMSC penetration depth following fibrous side seeding was approximately 150 μm after 4 d culture.

### Effect of BP ECM niche on seeded hMSC

**Figure 2:**
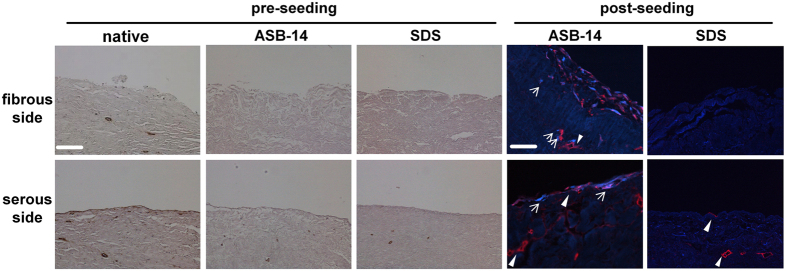
Immunohistochemistry staining of laminin on BP scaffold and immunofluorescent staining of laminin (red) and eGFP (blue) on seeded BP scaffold (200×). Laminin is present on the serous side of native BP, but absent on the fibrous side. ASB-14 scaffolds largely preserve laminin presence, whereas SDS scaffolds demonstrate disruption of laminin architecture. Following seeding, hMSC on the fibrous side of ASB-14 scaffolds are positive for laminin co-staining. No cells are evident on SDS scaffolds. Arrows indicates hMSC. Scale bar represents 100 μm.

Positive staining for laminin was observed on the serous side of native BP and ASB-14 scaffolds in a contiguous pattern along the tissue surface and around vessels within the tissue (Fig. 2). In SDS scaffolds, laminin was rarely and discontinuously observed on the serous surface, but still present around vasculature. There was no laminin on the fibrous surface of either native BP or BP scaffolds.

Positive staining for eGFP was observed only in ASB-14 scaffolds. Following seeding on the fibrous side, cells were found both on the surface and penetrating deep into the scaffold. Furthermore, laminin, not observed in non-seeded ASB-14 scaffolds, was detected around hMSC both at the surface and deeper within ASB-14 scaffolds (Fig. 2). Following seeding on the serous side, cells formed a confluent monolayer, and did not invade into the deeper layers of the scaffold (Fig. 2). The same growth patterns were observed when using different cell-seeding densities on both surfaces of ASB-14 scaffolds.

### Morphology of fibrous and serous surfaces on ASB-14 scaffolds pre and post-seeding

**Figure 3:**
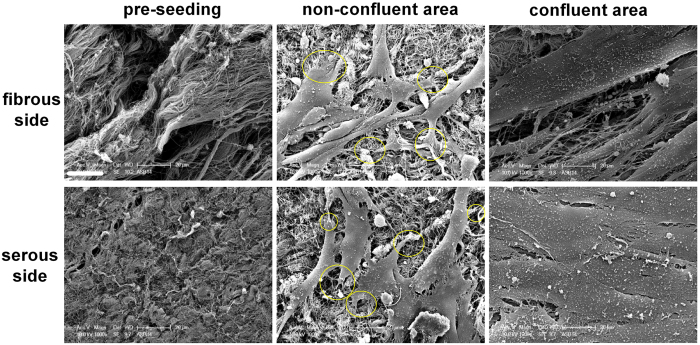
Scanning electron microscope (SEM) images of ASB-14 scaffolds before and after cell seeding (1000×). ASB-14 scaffolds possess a distinct sidedness. Collagen forms thicker bundles on fibrous side, while oriented collagen fibers on the serous side forming a smooth surface. Circles indicate the attachment points of cells to the material surface. The sidedness is no longer present following seeding. Scale bar represents 20 μm (n = 4).

Prior to recellularization, thicker collagen bundles were observed on fibrous side of ASB-14 scaffolds while the serous side consisted of a relatively smooth surface of aligned collagen fibers (Fig. 3). In non-confluent regions on both sides of ASB-14 scaffolds, hMSC spreading was observed with finger-like cellular projections interacting with scaffold components (Fig. 3). The scaffold sidedness prior to seeding was no longer present following recellularization (Fig. 3). After 4 d cultivation, cells formed a confluent monolayer on both sides of the scaffold.

### Effect of ECM niche on maximal hMSC seeding density and binding kinetics

**Figure 4:**
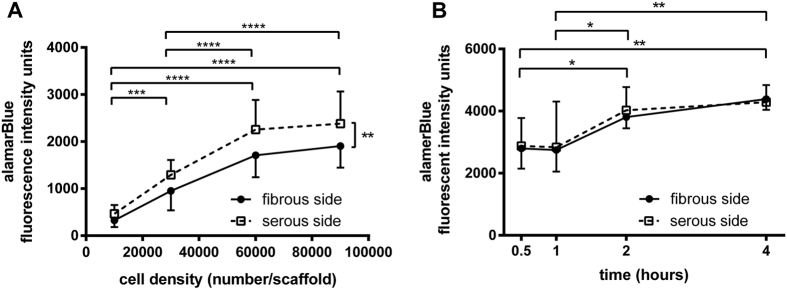
Maximal seeding density and binding kinetics for hMSC on ASB-14 scaffolds. (**A**) Adhesion of viable cells increases with cell seeding up to 60,000 cells/scaffold (***p < 0.005, n = 7 per group). Serous side supports a greater density of hMSC compared to the fibrous side (**p < 0.01). (**B**) No significant difference in kinetics of hMSC adhesion to BP ECM is found between fibrous and serous seeding (n = 4 per group/time point). Regardless of seeding location, number of adherent cells increases until 2 h of contact time (*p < 0.05). Data are shown as the mean ± standard deviation.

A loading density-dependent increase in adhesion of viable hMSC was found up to 60,000 cells/scaffold regardless of seeding side on ASB-14 scaffolds (Fig. 4A, *p* < 0.005). Above this seeding density, no further increase in viable cell adhesion was achieved (*p* > *0*.*05*). A higher number of adherent hMSC was supported by the serous side compared to the fibrous side (*p* = 0.0032). Following determination that at 30,000 cells/scaffold the seeding efficiency was not different between sides (*p* = 0.1109), this seeding density was used to test how rapidly cells bind on each side of ASB-14 scaffolds. The number of adherent cells did not differ during the first hour of contact (*p* > *0*.*05*), but increased from 1 h to 2 h (*p* < 0.05) and reached steady state after 2 h (Fig. 4B, *p* > *0*.*05*). No significant difference in seeding kinetics was found between fibrous and serous sides (*p* = 0.7903).

### Effect of ECM niche on hMSC proliferation

**Figure 5:**
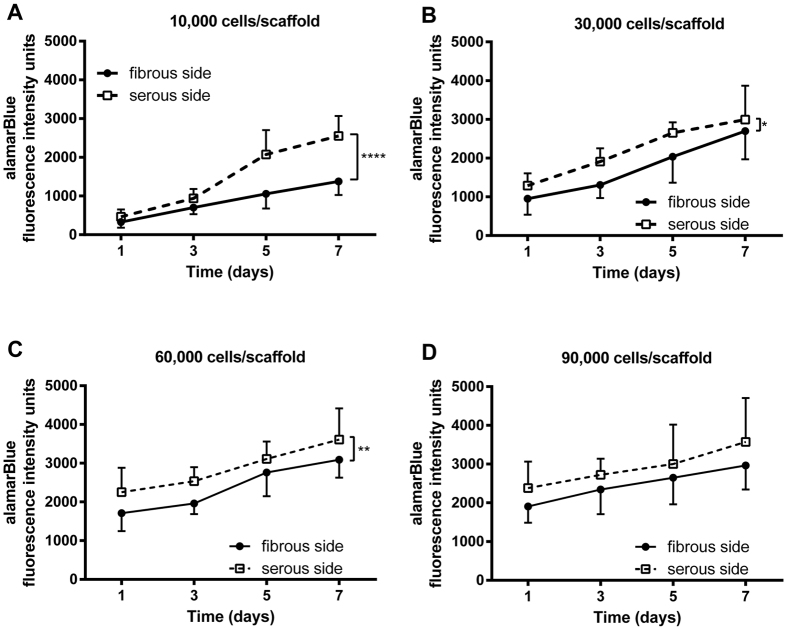
hMSC proliferation on ASB-14 scaffolds at increasing seeding densities. (**A**) 10,000 cells/scaffold; (**B**) 30,000 cells/scaffold; (**C**) 60,000 cells/scaffold; and (**D**) 90,000 cells/scaffold. After 7 d cultivation, cells significantly expand at all cell densities. Greater cell proliferation occurs following serous seeding than for fibrous seeding for initial seeding densities of between 10,000 and 60,000 cells/scaffold. Data are shown as the mean ± standard deviation (*n* = 7 per group, *p < 0.05, **p < 0.01, ****p < 0.001).

Regardless of initial seeding density or side, hMSC proliferated after 7 d cultivation on ASB-14 scaffolds (Fig. 5, *p* < 0.0001). hMSC proliferated more rapidly on the serous side compared to fibrous side from 10,000 (*p* < 0.0001) to 60,000 cells/scaffold (*p* = 0.0047), but this difference was no longer present at 90,000 cells/scaffold (*p* = 0.1632).

### Tri-lineage differentiation potential of hMSC after cultivation on ASB-14 scaffolds

Effective hMSC differentiation into adipogenic, osteogenic, and chondrogenic lineages was observed for cells following seeding on both fibrous and serous sides of ASB-14 scaffolds (Supplemental Fig. 1).

### Effect of different ECM culture environments on hMSC HLA expression

**Figure 6:**
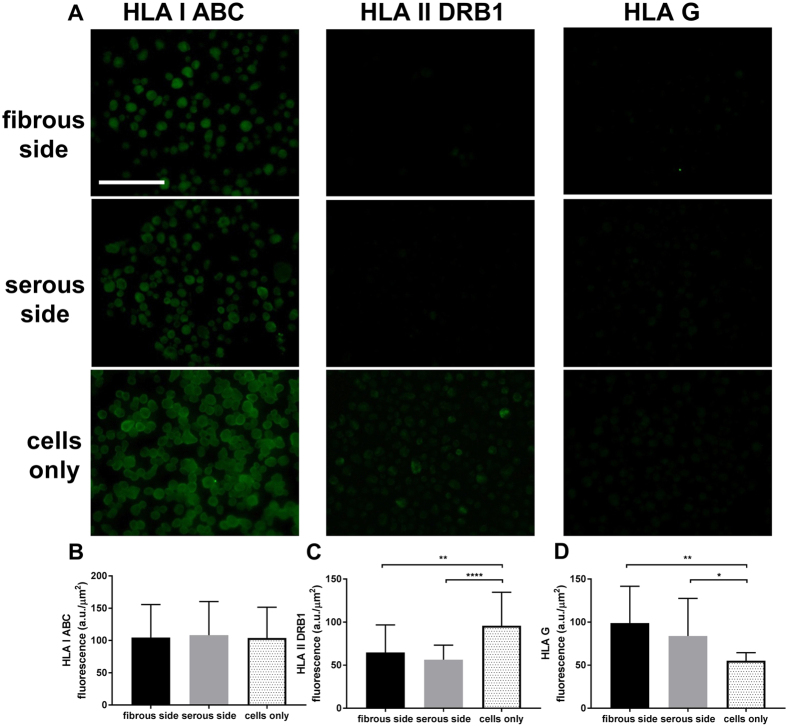
Immunofluorescent staining of HLA class I ABC, HLA class II DRB1 and HLA G proteins in hMSC seeded on fibrous or serous side of ASB-14 scaffold, and tissue culture plastic. (**A**) HLA class I ABC proteins were detected in all the MSCs under the different seeding conditions. HLA class II DRB1 was weakly stained in hMSC on plastic, and further reduced in hMSC on ASB-14 scaffolds. HLA G staining was low in hMSC on plastic, and increased following seeding on ASB-14 scaffolds. Scale bar represents 100 μm. (**B**) No statistically significant difference was observed on HLA class I ABC expression between groups (*p* > 0.05). (**C**) Cells on fibrous side (*p* = 0.0026) and serous side (****p < 0.0001) express significantly less HLA class II DRB1 compared to cells on tissue culture plastic. (**D**) Cells on fibrous side (*p* = 0.0014) and serous side (*p* = 0.0187) express significantly more HLA G compared to cells on tissue culture plastic. Fluorescent intensity is expressed as arbitrary unit (a.u.)/μm^2^. Data are shown as the mean ± standard deviation (n = 20–30 cells per stain per group).

The HLA class I ABC proteins were relatively strongly stained in all hMSC after 3 d of culture either on tissue culture plastic or ASB-14 scaffold regardless of seeding side (Fig. 6A). Weak HLA class II DRB1 and HLA G staining were detected (Fig. 6A). No significant difference was observed for hMSC HLA class I ABC expression among cells seeded on fibrous (108.300 ± 50.662 a.u./μm^2^), or serous (111.166 ± 51.643 a.u./μm^2^) surface compared to tissue culture plastic (105.960 ± 47.916 a.u./μm^2^) seeding (Fig. 6B) (p > 0.05). Seeding on the serous side of ASB-14 scaffolds (55.970 ± 17.314 a.u./μm^2^) (*p* < *0*.*0001*) or fibrous side seeding (64.760 ± 33.644 a.u./μm^2^) (*p* = 0.0026) significantly decreased HLA II DRB1 compared to cells on tissue culture plastic (97.787 ± 38.772 a.u./μm^2^) (Fig. 6C). hMSC HLA G expression on fibrous side (99.687 ± 44.080 a.u./μm^2^) (*p* = 0.0014) and serous side (84.515 ± 45.065 a.u./μm^2^) (*p* = 0.0187) significantly increased compared to cells on tissue culture plastic (55.042 ± 9.615 a.u./μm^2^) (Fig. 6D).

### Effect of different ECM culture environments on hMSC inflammatory cytokine production

**Figure 7:**
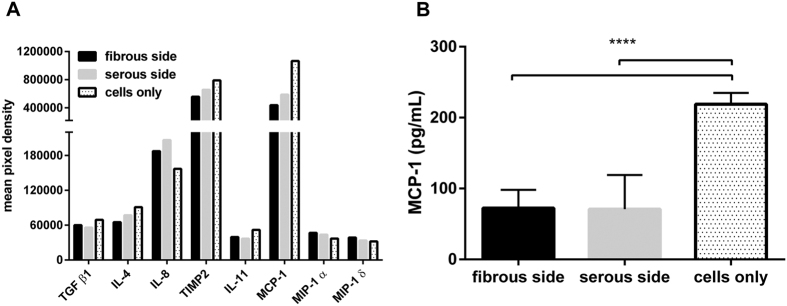
Human inflammation cytokines data. (**A**) Differential secretion of cytokines from hMSC on either side of ASB-14 scaffolds or on plastic: transforming growth factor beta 1 (TGF β1), monocyte chemoattractant protein-1 (MCP-1), interleukin 4 (IL-4), interleukin 8 (IL-8), interleukin 11 (IL-11), tissue inhibitors of metalloproteinases 2 (TIMP2), macrophage inflammatory protein 1 alpha and delta (MIP-1α and δ). Values represent mean chemiluminescent intensity measurements of duplicate dots of stained membranes (n = 1, conditioned medium from six replicates per group was pooled and assayed). (**B**) ELISA measurement of MCP-1 released from hMSC. MCP-1 concentration on ASB-14 scaffold is significantly lower than on culture plastic. Culture media and ASB-14 BP scaffold only samples are used as controls. Data are shown as the mean ± standard deviation (n = 6 per group, ****p < 0.001).

Of the 40 inflammatory cytokines assessed via protein microarray, 5 factors (TGF β1, IL-4, IL-11, TIMP2, MCP-1) were decreased following seeding on ASB-14 scaffolds and 3 were increased (IL-8, MIP-1 α and MIP-1δ) (Fig. 7). MCP-1 concentration in culture supernatant of hMSC on tissue culture plastic (218.878 ± 16.053 pg/mL) was significantly greater than that of hMSC on ASB-14 scaffolds (*p* < 0.001) (Fig. 7). However, no significant difference was observed for MCP-1 concentration between recellularized serous (70.806 ± 48.225 pg/mL) and fibrous (72.381 ± 25.729 pg/mL) ASB-14 scaffold surfaces (*p* > *0*.*05*).

### Effect of different ECM culture environments on hMSC angiogenic cytokine production

**Figure 8:**
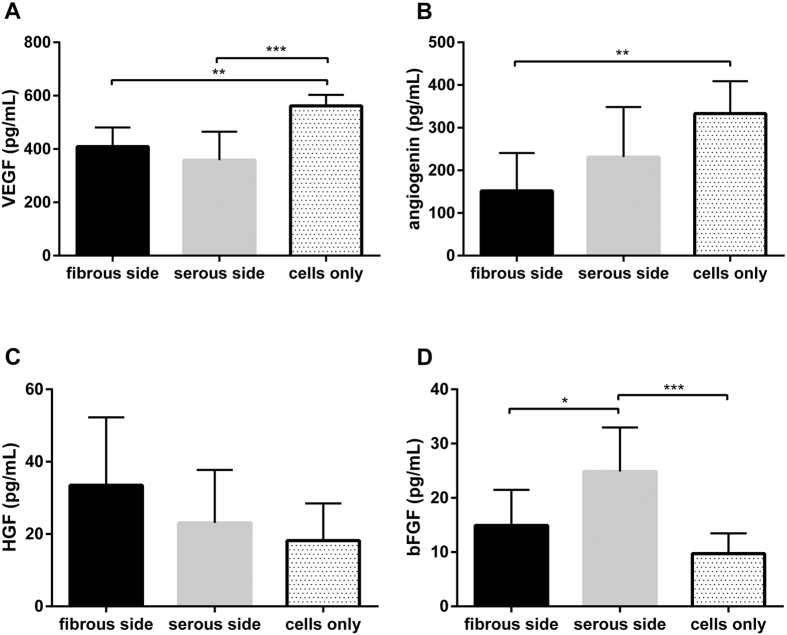
Human angiogenesis-related cytokines data. Differential secretion of cytokines from hMSC on either side of ASB-14 scaffolds or on plastic: (**A**) Vascular endothelial growth factor (VEGF) is highly secreted by hMSC on culture plastic compared to that on ASB-14 scaffolds. (**B**) Angiogenin secretion is lower when seeded on fibrous side of ASB-14 scaffolds compared to on culture plastic. (**C**) The difference of hepatocyte growth factor (HGF) among three groups is not apparent. (**D**) Basic fibroblast growth factor (bFGF). Cells on serous side of ASB-14 scaffolds release more bFGF than on the fibrous side and on the culture plastic. Data are shown as the mean ± standard deviation (n = 7 per group, *p < 0.05, **p < 0.01, ***p < 0.005).

Of the 10 angiogenic cytokines assessed in the microarray, 2 factors (VEGF and angiogenin) were downregulated following seeding on ASB-14 scaffolds and bFGF was upregulated. VEGF concentration in culture supernatant of hMSC on tissue culture plastic (561.839 ± 41.400 pg/mL) was significantly greater than that of hMSC on ASB-14 scaffolds (*p* < 0.01) (Fig. 8A). However, no difference in VEGF concentration was found between recellularized serous (358.254 ± 107.077 pg/mL) and fibrous (409.237 ± 72.092 pg/mL) ASB-14 scaffold surfaces (*p* > *0*.*05*). No difference was found in angiogenin production between recellularized serous (231.089 ± 117.248 pg/mL) and fibrous (151.731 ± 88.853 pg/mL) (*p* > *0*.*05*) ASB-14 scaffold surfaces, although angiogenin concentration was higher with tissue culture plastic (333.115 ± 75.671 pg/mL) than on the fibrous side (*p* < 0.01) (Fig. 8B). Although HGF concentrations from cells seeded on either the serous or fibrous side (23.095 ± 14.617 or 33.458 ± 18.835 pg/mL, respectively) trended higher compared to those on tissue culture plastic (18.165 ± 10.334 pg/mL), this finding was not significant (*p* > *0*.*05*) (Fig. 8C). bFGF concentration was significantly increased following seeding on the serous side (24.857 ± 8.120 pg/mL) compared to on plastic (9.732 ± 3.717 pg/mL) or the fibrous side (14.931 ± 6.560 pg/mL) (*p* < 0.05) (Fig. 8D).

## Discussion

The present study demonstrates the critical importance of scaffold generation method and resultant ECM niche preservation on cellular repopulation and cellular functional properties. Specifically, we found that (1) SDS-decellularization results in BP scaffolds which are toxic toward hMSC, resulting in inability of the cells to bind and/or be maintained on the scaffold. (2) Scaffolds generated using ASB-14 are capable of supporting cell binding, migration, and proliferation. (3) Growth pattern and function of hMSC is affected by the ECM niche onto which the cells are seeded in ASB-14 scaffolds. (4) Following seeding on ASB-14 scaffolds, hMSC retain their tri-lineage differentiation potential. (5) ASB-14 scaffold sidedness can significantly alter hMSC production of several angiogenic and inflammatory cytokines. (6) ASB-14 ECM niche differentially affects seeded hMSC HLA expression. These findings may have important implications for the generation, cellular repopulation and use of xenogeneic ECM scaffolds in clinical practice.

Residual toxicity of an ECM scaffold is a critical consideration in development of such biomaterials for potential clinical applications^32^,^33^. Here, we demonstrate that although ASB-14 and SDS are equally toxic to hMSC, scaffolds generated with ASB-14 are non-toxic following 4 d of washout and are therefore capable of supporting hMSC adhesion and proliferation. Conversely, SDS scaffolds continue to leach components which are toxic to 96.5% of cells, even after 7 d of washing. Previous reports of the effects of SDS-decellularization on resultant scaffold recellularization potential have shown mixed results, with some groups showing positive findings and others describing negative results^32^,^33^,^34^,^35^,^36^,^37^,^38^,^39^,^40^,^41^,^42^,^43^,^44^. The possible reasons for the differences in recellularization potential reported for SDS-decellularized scaffolds include: (1) Different tissues and organs may alter the ability to remove SDS from resultant scaffolds. (2) Different washing methods are used in SDS-decellularization approaches. (3) Sensitivity of different cell types to SDS toxicity may vary. Although the current study utilized the longest washout time reported to date for SDS-decellularized tissue (14 washing cycles, totaling 28 mL of washing solution for 0.2 g tissue), resultant scaffolds remained toxic to essentially all reseeded hMSC. The highly collagenous nature of BP compared to other tissues may result in high levels of SDS binding and resultant toxicity^18^,^44^. To overcome the potential for residual toxicity following SDS-decellularization, low concentration and rigorous washing steps have been recommended^45^. However, even with use of a low SDS concentration (0.1%) to generate porcine pulmonary or aortic root scaffolds, Rieder *et al*. found no human saphenous vein endothelial cell attachment took place and massive cell lysis was observed^36^. Alteration in pH or washing with Triton X-100 has been reported to be effective for removal of residual SDS in some tissue types^45^. Grazer *et al*. found that using pH 9 tris-buffer successfully decreased residual SDS concentration in porcine anterior cruciate ligament scaffold, but low autologous fibroblasts repopulation was still observed in SDS group due to the alterations in tissue matrix biochemistry and structure by extreme wash conditions^38^. Similarly, Syed *et al*. failed to improve the poor biocompatibility (~10% of viable human esophageal smooth muscle cells) of small intestine submucosa scaffolds generated by SDS-decellularization when including a Triton X-100 wash method^43^. Moreover, different cell types tolerate SDS toxicity differently. Cebotari *et al*. reported the residual 0.005% SDS was toxic to 17% of human endothelial cells^32^, while 0.0048% is the LD_50_ of hMSC to SDS reported here, indicating that hMSC are particularly sensitive to the toxic effects of SDS. Regardless of the mechanism for the residual toxicity of SDS-decellularized scaffolds demonstrated here, we conclude that SDS-decellularization may not be an appropriate method for formation of BP ECM scaffolds. Conversely, the ability of ASB-14 scaffolds to be rendered non-toxic following relatively short wash periods suggests that such scaffolds may be appropriate for clinical use.

The anatomy of BP is comprised of two distinct, but inseparable biologic surfaces: the fibrous pericardial layer facing the thoracic cavity and the serous parietal pericardial layer facing the epicardium. The fibrous surface is composed of a loose arrangement of collagenous and elastic fibers (loose connective tissue), while the serous surface is composed of basement membrane supporting the mesothelial cell monolayer. When such biomaterials are exposed to blood flow, sidedness has potential to influence host response towards the biomaterial. Pries *et al*. reported the risk of graft calcification increases when the fibrous side was in contact with blood flow, and large functional differences were demonstrated between the serous and fibrous sides when BP was used as a patch for arterial closure^46^. Additionally, Gauvin *et al*. showed that exposure of the fibrous side to the vascular lumen results in increased platelet adhesion and exacerbated acute thrombogenicity compared to serous side^47^. Here we studied hMSC behavior on different sides of ASB-14 scaffolds. We found both sides of ASB-14 scaffolds maintain the suitable microenvironment for relatively high levels of cell attachment and a comparable proliferation rate to cells on 96-well plate (data not shown). On average, cells grown on ASB-14 scaffolds increased by 4.8-fold and 1.4-fold when seeded at 10,000 cells/well and 90,000 cells/well within 7 d cultivation, respectively. Furthermore, positive laminin staining (Fig. 2) demonstrated that an intact basement membrane complex was preserved on serous side. Basement membrane is a critical component of the ECM that supports and facilitates the growth of cells^12^,^48^. Its distinct surface chemistry may modulate focal adhesion composition and signaling through changes in integrin binding, and different adhesive interactions activate various intracellular signaling pathways that direct cell cytokines release^49^. Here we demonstrate the ability of the preserved ECM niche environment to direct site-specific cell behavior: monolayer formation on the serous side and ingrowth on the fibrous surface (Fig. 2), similar to other sided materials, such as porcine urinary bladder scaffold^48^. Furthermore, seeding on the serous surface resulted in increased bFGF, a factor commonly associated with maintenance of “stemness” by stem cells^50^, compared to fibrous side seeding (Fig. 8). Such high cell retention and sidedness properties of ASB-14 scaffolds may have important clinical implications. For instance, cardiac therapies using stem cells have typically been hindered by poor retention of transplanted cells^51^. If migratory ability of both delivered and endogenous cells is desirable (e.g., cardiac repair), the fibrous side should be positioned to facilitate such cellular repopulation and retention. Alternatively, the ability of serous side to support a higher number of cells (Fig. 4) and form a monolayer may be critical in applications where such a layer is expected to be beneficial (e.g., endothelial monolayer formation^52^). Acute thrombosis occurs with exposure of foreign tissue to host blood. By promoting quicker cell monolayer formation, together with the increased secretion of bFGF, pre-seeding of the serous surface with hMSC with high differentiation potential may have potential to increase recruitment of endogenous cells and/or reduce thrombogenicity. However, clinically relevant benefits of such sidedness properties but will require further investigation for validation. Besides distinct sidedness-based niches, we demonstrate that ASB-14 scaffolds can significantly alter hMSC cytokine secretion profile compared to cells on plastic. Of the 40 inflammatory proteins, one important pro-inflammatory cytokine MCP-1 was significantly downregulated. MCP-1 is the key chemokine for macrophage recruitment^53^. Decreased MCP-1 would bring less antigen-presenting cells to the site which has the potential to further modulated antigenicity of the material. However, of the other analyzed cytokines three pro-inflammatory factors (IL-8, MIP-1α and MIP-1δ) trended to be increased and four anti-inflammatory factors (TGF β1, IL-4 TIMP2 and IL-11) trended to be decreased. Meanwhile decreased VEGF and angiogenin levels were also observed. Furthermore, we demonstrate that ASB-14 scaffolds can affect hMSC HLA expression patterns compared to cells on plastic. HLA system, the human version of the major histocompatibility complex (MHC), plays a critical role in regulating the graft-versus-host immune response. Here we found that seeding on ASB-14 scaffolds increased hMSC HLA-G expression, a crucial immunosuppressive protein by 1.81 (fibrous side) and 1.54 fold (serous side) compared to cells grown on tissue culture plastic. Scaffold seeded resulted in decreased in expression of pro-inflammatory HLA class II markers by 1.51 (fibrous side) and 1.74-fold (serous side) compared to cells on plastic. Together with no influence on HLA class I ABC expression, hMSC on ASB-14 scaffold represent potentially promising immunomodulatory stem cells for *in vivo* applications. Future investigations will be necessary to determine the predominant mechanism (e.g., ECM architecture vs. composition vs. mechanics) responsible for inducing such differential cytokine and HLA production, as well as whether differential cytokine release has additional effects on HLA expression as other studies reported^54^,^55^. *In vivo* studies will also be required to examine the combined influence of such cytokine and HLA changes on immunomodulatory and proangiogenic potential of hMSC seeded ASB-14 scaffolds.

Over decades of research, there have only been limited studies on structural and functional variations of pericardial tissue between distinct sides^46^,^47^,^56^,^57^. Here, we compared the release of pro- and anti- inflammatory cytokines, angiogenesis related cytokines, and HLA expression by hMSC subjected to differential sidedness-based pericardial niches. Important differences were identified in cellular behavior (e.g., maximal seeding density, proliferation rate, migration, cytokines profile, and HLA expression) following fibrous versus serous seeding, adding to our knowledge of sidedness-based properties and draw attention to its importance when using such tissue to generate scaffolds. However, the differences we found in the cytokine release and HLA expression were based on bone marrow derived MSC from one donor. Source to source, or donor to donor variability for MSC has not been quantitatively assessed here. Continued research with different MSC phenotypes from different donors and source tissues (e.g., adipose derived) are warranted^58^.

## Conclusion

This study demonstrates that ECM scaffold preparation method critically effects recellularization potential. Scaffolds generated using 1% SDS were toxic to repopulating cells, while those generates using 1% ASB-14 were compatible with cellular repopulation. Additionally, preservation of the ECM niche in ASB-14 scaffolds resulted in differential modulation of cellular behavior (e.g. migration, maximal seeding density, proliferation rate) and function (e.g., cytokine release, HLA expression) dependent on seeding location. Future work is necessary to determine the predominant mechanism responsible for sidedness dependent differential cytokine and HLA expression, and how such effects on cellular behavior may result in differential *in vivo* response. As tissue and organ engineering efforts become more sophisticated, these findings improve our understanding of the interaction of cells and matrix and indicate the ASB-14 scaffolds may hold great potential for regenerative applications.

## Additional Information

**How to cite this article**: Liu, Z. Z. *et al*. Effect of bovine pericardial extracellular matrix scaffold niche on seeded human mesenchymal stem cell function. *Sci. Rep*. **6**, 37089; doi: 10.1038/srep37089 (2016).

**Publisher’s note:** Springer Nature remains neutral with regard to jurisdictional claims in published maps and institutional affiliations.

## Supplementary Material

Supplementary Information
