# Supplementary material for: Effect of bovine pericardial extracellular matrix scaffold niche on seeded human mesenchymal stem cell function
Source: Sci Rep. 2016 Nov 15;6:37089. doi: 10.1038/srep37089 (PMC5109049; doi:10.1038/srep37089)
Supplement: Supplementary Information [file srep37089-s1.pdf]

Effect of bovine pericardial extracellular matrix scaffold niche on seeded human mesenchymal stem cell function.

Running headline: Extracellular matrix niche effects stem cell function

Zhi Zhao Liu, Maelene L. Wong, Leigh G. Griffiths*

Department of Veterinary Medicine: Medicine and Epidemiology, University of California, Davis, One Shields Ave., Davis, CA 95616, USA

*Corresponding author: Department of Medicine and Epidemiology, University of California, Davis, One Shields Avenue, Davis, CA, 95616, USA. Tel: +1 530 754 0334

Email address: lggriffiths@ucdavis.edu (Leigh G. Griffiths)

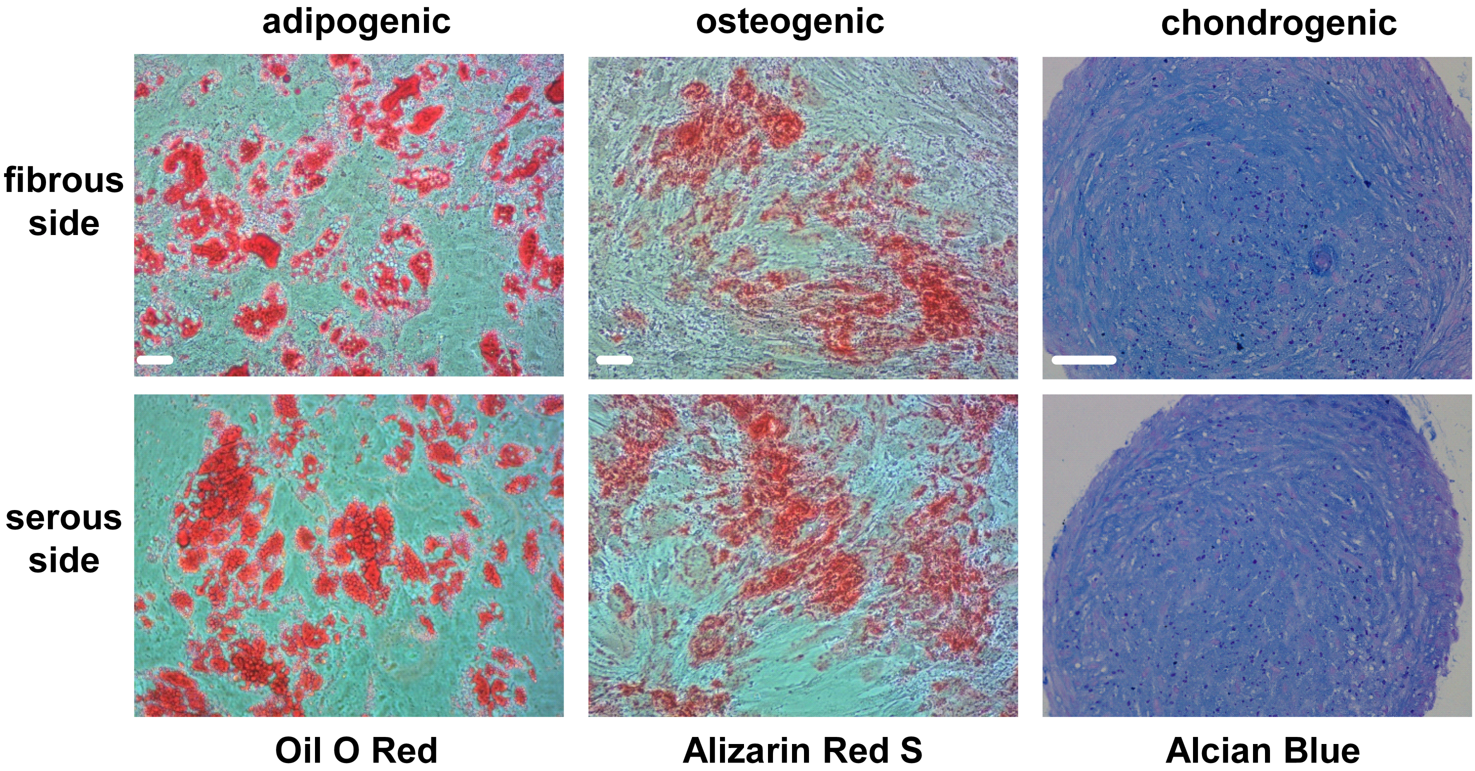
adipogenic
osteogenic
chondrogenic
fibrous side
serous side
Oil O Red
Alizarin Red S
Alcian Blue

Supplementary Fig. 1: Trilineage differentiation of hMSC retrieved from ASB-14 scaffolds. Representative microscopic images of Oil Red O staining and Alizarin Red (100×) to confirm adipogenic and osteogenic differentiation of hMSC. Chondrogenic differentiation is confirmed by the formation of cell pellets that were positive for Alcian Blue (200×). Scale bar represents 100 µm. Upper row: induced hMSC detached from the fibrous side of ASB-14 scaffolds; lower row: induced hMSC detached off the serous side of ASB-14 scaffolds (n=5 per group).
